# Comparison of Azithromycin Removal from Water Using UV Radiation, Fe (VI) Oxidation Process and ZnO Nanoparticles

**DOI:** 10.3390/ijerph17051758

**Published:** 2020-03-08

**Authors:** Amirreza Talaiekhozani, Sahar Joudaki, Farhad Banisharif, Zeinab Eskandari, Jinwoo Cho, Ghasem Moghadam, Shahabaldin Rezania

**Affiliations:** 1 Department of Civil Engineering, Jami Institute of Technology, PO 8491963395, Isfahan, Iran; atalaie@jami.ac.ir; 2 Department of Chemical Engineering, Jami Institute of Technology, PO 8491963395, Isfahan, Iran; sahar.joudaki1366@gmail.com (S.J.); zeinabeskandari@jami.ac.ir (Z.E.); 3 School of Chemical, Petroleum and Gas Engineering, Iran University of Science and Technology, P.O. Box 16675-163, Narmak, Tehran, Iran; f.banisharifdehkordey@gmail.com; 4 Department of Environment and Energy, Sejong University, Seoul 05006, Korea; jinwoocho@sejong.ac.kr; 5 Faculty of Science, Islamic Azad University of Shahrekord branch, P.O. Box 166, Shahrekord, Iran; moghadam.ghasem@gmail.com; 6 Young Researchers and Elite Club, Shahrekord Branch, Islamic Azad University, P.O. Box 166, Shahrekord, Iran

**Keywords:** Fe (VI) oxidation process, UV radiation, ZnO nanoparticles, azithromycin removal, wastewater treatment, aquatic antibiotic solution

## Abstract

Antibiotics are resistant to biodegradation, and their removal by biological processes is difficult. The purpose of this study was to investigate the removal of azithromycin from water using ultraviolet radiation (UV), Fe (VI) oxidation process and ZnO nanoparticles. The effect of different parameters such as pH, temperature, hydraulic retention time (HRT), the concentration of Fe (VI) and ZnO nanoparticles and UV intensity on the removal of azithromycin from water was investigated. The optimal conditions for the removal of azithromycin were a pH of 2, a temperature of 25 °C, a HRT of 15 min, and a ratio of ZnO nanoparticles to the initial concentration of azithromycin (A/P) of 0.00009 which was fitted by Langmuir isotherm. In addition, the optimal conditions for the removal of azithromycin using UV radiation were a pH of 7, a temperature of 65 °C, a HRT of 60 min, and UV radiation power of 163 mW/cm^2^. For the Fe (VI) oxidation process, the optimal conditions were a pH of 2, a temperature of 50 °C and a HRT of 20 min. Also, the optimal ratio of Fe (VI) to the initial concentration of antibiotic was between 0.011 and 0.012. The results of this study showed that the Fe (VI) oxidation process, UV radiation, and ZnO nanoparticles were efficient methods for the removal of azithromycin from water.

## 1. Introduction

Nowadays, with the growing population and the expansion of industry, the need for wastewater treatment has become important [1,2]. Wastewater contains several pollutants which are harmful to human and animal health [3]. Pharmaceutical industries produce wastewater containing a wide range of recalcitrant chemicals such as antibiotics that can lead to several environmental issues [4,5]. Antibiotics account for about 15% of the total drugs produced worldwide and cause the death of microorganisms in wastewater treatment in biological systems such as activated sludge and biotrickling filters [6]. The presence of antibiotics in drinking water increases the resistance of pathogenic microorganisms to antibiotics [7]. There are many ways to release antibiotics into water sources such as pharmaceutical industries and human as well as animal stools. Antibiotics are excreted into the wastewater after being used for the treatment of infectious diseases in humans and animals through their feces. Depending on the animal species and the type of antibiotic, between 30% and 90% of antibiotics added to animal foods are excreted; a part of this can be released into wastewater [8]. Wei et al. studied the presence of various antibiotics in wastewater and surface water around poultry farms and large-scale livestock in Jiangsu Province, China [9]. They identified sulfamethoxazole, sulfadiazine, tetracycline, sulfamethazine and oxytetracycline as the most predominant antibiotics in that area, with concentrations of 63.6, 17.0, 10.3, 72.9, and 211 μg/L, respectively [9]. Yang et al. reported the presence of many antibiotics such as sulfadimethoxine, sulfamethoxazole, sulfachloropyridazine, sulfamethazine, sulfathiazole, meclocycline, doxycycline, chlortetracycline, demeclocycline, tetracycline and oxytetracycline in raw wastewater and treated domestic wastewater [10]. Bhandari et al. reported that the average concentrations for azithromycin, sulfamethoxazole and ciprofloxacin in the raw wastewater in the United State of America were 18.3, 1.11 and 1.44 μg/L, respectively [11]. They also reported that the concentrations of azithromycin, sulfamethoxazole and ciprofloxacin in the treated wastewater were 3.25, 1.23, and 0.59 μg/L, respectively. Other reports have shown that azithromycin can be found in domestic wastewater [12]. These data show that conventional biological systems for domestic wastewater treatment are not able to remove all antibiotics from wastewater.

**Figure 1:**
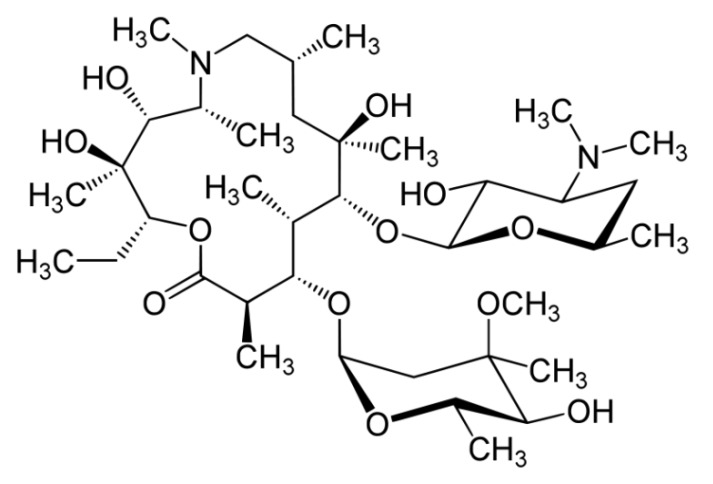
Molecular structure of azithromycin.

Azithromycin is an antibiotic which is widely used in the treatment of bacterial infections such as streptococcal pharyngitis, pneumonia, diarrhea and intestinal inflammation [13]. Antibiotics are not biodegradable but they can be removed from aquatic solutions by physicochemical treatment systems such as electrocoagulation [14], osmotic membrane bioreactor [15] and chemical oxidation processes [6,16,17]. The molecular structure of azithromycin is shown in Figure 1.

Among the various methods of antibiotic removal from aquatic solutions, the use of oxidants such as chlorine [18], ozone [19], chlorine dioxide [20], advanced oxidation methods [21] and Fe (VI) [22] is the most common. Chlorine is a low cost oxidant that can remove antibiotics efficiently, but can also produce dangerous byproducts such as trihalomethanes [23], making it unsuitable for the removal of azithromycin from aquatic solutions. Although oxidizers such as ozone and chlorine dioxide do not produce byproducts, they are expensive. Fe (VI), one of the most powerful oxidizers in acidic conditions, can be produced at low cost, and its usage for the removal of pollutants from aquatic solutions has been considered recently [24]. Fe (VI) is gradually converted to Fe (III), which is a powerful coagulant. Fe (VI) can oxidize organic compounds such as DNA; therefore, it is also considered as a disinfectant [25]. The process of chemical oxidation, coagulation and flocculation, as well as disinfection, can be performed in one unit when a Fe (VI) oxidation process is used [26].

UV can degrade the molecules of organic compounds, yielding simpler molecules; therefore, many attempts have been made to use UV for wastewater treatment. Additionally, UV radiation may enhance the activity of photocatalysts such as MgO, ZnO and TiO_2_ [27]. It also used as an activator for several advanced oxidation processes, such as the Fenton process. Thus, the simultaneous usage of UV with other chemical processes can increase the efficacy of the removal of pollutants from water. In this study, the UV radiation method was used for the removal of azithromycin from an aquatic solution [28]. Adsorption is another method used to remove pollutants from aquatic solutions [29,30]. Various absorbents have been introduced for wastewater treatment [31], such as ZnO nanoparticles. Although ZnO nanoparticles can adsorb pollutants from water, they can act as a photocatalyst under UV radiation. In this study, the ability of ZnO nanoparticles to remove azithromycin from a synthetic aquatic solution was also investigated.

There is no published study on the removal of azithromycin from aquatic solutions with Fe (VI) oxidation process, UV radiation and ZnO nanoparticles. Therefore, the objectives of this study were (a) to investigate the ability of the Fe (VI) oxidation process, UV radiation and ZnO nanoparticles to remove azithromycin from a synthetic aquatic solution, and (b) to investigate the effects of pH, temperature and hydraulic retention time (HRT) on these three methods.

## 2. Materials and Methods

### 2.1. Synthetic Aquatic Solution Contaminated with Azithromycin (ASCA)

To produce ASCA, 2 g of azithromycin was added to 1000 mL of distilled water. The solution was then kept in a refrigerator at 4 °C for 24 h, before being passed through filter paper separate out suspended parts of the antibiotic. Then, the chemical oxygen demand (COD) of filtered ASCA was measured.

### 2.2. ZnO Nanoparticles

#### 2.2.1. Effect of pH

Four Erlenmeyer flasks containing 100 mL of synthetic ASCA were prepared. Next, different amounts of hydrochloric acid or sodium hydroxide was added to the Erlenmeyer flasks to obtain pHs of 2, 5, 9 and 12. Then, 0.05 mg/L ZnO nanoparticles were added to each of the flasks. Next, the Erlenmeyer flasks were kept at room temperature at 25 °C for 15 min. In the end, the concentration of COD in each Erlenmeyer flask was determined.

#### 2.2.2. Effect of ZnO Nanoparticles

Five Erlenmeyer flasks with a volume of 250 mL containing 10 mL of synthetic ASCA were prepared. Then, a suitable amount of hydrochloric acid (HCl) was added to adjust the pH on 2. Next, an appropriate amount of ZnO nanoparticles was added to flasks 1, 2, 3, 4 and 5 to reach concentrations of 0.01, 0.02, 0.03, 0.04 and 0.05 mg/L, respectively. After that, the flasks were kept at room temperature at 25 °C for 15 min.

#### 2.2.3. Effect of HRT

The effect of HRT on antibiotic removal by ZnO nanoparticles was investigated in four different HRTs of 5, 10, 15 and 20 min. In this set of experiments, the pH was adjusted to 2, the concentration of ZnO nanoparticles was 0.05 mg/L and the temperature was 25 °C.

#### 2.2.4. Effect of Temperature

A flask containing 100 mL of synthetic ASCA was prepared. Then, the pH of synthetic ASCA was adjusted to 2 by adding a suitable amount of hydrochloric acid. The concentration of ZnO nanoparticles was adjusted to 0.05 mg/L. After that, the flask was lept at 25 °C for 15 min. Next, the COD concentration of the flask was measured. This procedure was repeated at temperatures of of 40, 60 and 90 °C.

#### 2.2.5. Determination of Isotherms

In this study, the isotherms of Freundlich, Langmuir, D-R, Jovanovic and Generalized were evaluated. Equation (1) shows the linearized Freundlich isotherm.

(1) $$\log\left(\frac{x}{m}\right)=logK_{f}+\frac{1}{n}\log\left(C_{e}\right)$$

where, *x/m* is the amount of dye adsorbed per weight of the adsorbent under equilibrium condition, *C_e_* is the equilibrium concentration of the adsorbed dye in the solution after the adsorption process, *n* and *K_f_* are the constant coefficients of the Freundlich equation. Equation (1) is similar to the general equation of a straight line (*y = ax + b*). The amount of *K_f_* and *n* can be calculated by plotting *log*(*x/m*) versus *log*(*C_e_*). Langmuir isotherm is shown in Equation (2).

(2) $$\frac{C_{e}}{\left(x/m\right)}=\frac{1}{q_{m}K_{e}}+\frac{1}{q_{m}}C_{e}$$

where *q_m_* and *K_e_* are Langmuir constants. This equation is similar to the general equation of a straight line (*y = ax + b*). The values of *a* and *b* can be determined by plotting *C_e_*/((*x/m*)) versus *C_e_*. The Temkin isotherm is shown in Equation (3). The constants $K_{T}$ and *B*_1_ can be calculated using a linear plot of x/m versus $\ln\left(C_{e}\right)$.

(3) $$x/m\;=B_{1}\ln\left(K_{T}\right)+B_{1}\ln\left(C_{e}\right)$$

where $K_{T}$ is the equilibrium binding constant (in L/mg) corresponding to maximum binding energy and the value increased with an increase in temperature for both the adsorbents, which is suggestive of the corresponding increase of maximum binding energy. Constant *B_1_* is related to the heat of adsorption [21]. The D-R isotherm, apart from being an analogue of the Langmuir isotherm, is more general than the Langmuir in that it rejects the homogenous surface or constant adsorption potential. The D-R isotherm is shown in Equation (4).

(4) ln(xm)=lnqmax−βε2

where *q_max_* is D-R constant and *ε* can be calculated using Equation (5).

(5) $$\varepsilon =RTln\left(1+\frac{1}{C_{e}}\right)$$

where *q_max_* is the maximum amount of adsorbate that can be adsorbed on the adsorbent, *B* is the constant related to energy, *C_e_* is the equilibrium concentration (in mg/L), *R* is a universal gas constant that is equal to 8.314 J/mol.K and *T* is the temperature (in Kelvin). The Generalized isotherm is shown in Equation (6).

(6) ln[(qmaxxm)−1]=ln(KG)−Nln(Ce)

where *K_G_* is the saturation constant (in mg/L), *N* is the cooperative binding constant, *q_max_* is the maximum adsorption capacity of the adsorbent (in mg/g), xm (in mg/g) and *C_e_* (in mg/L) are the equilibrium dye concentrations in the soil and liquid phase, respectively. The values of *N* and *K_G_* are calculated from the slope and intercept of the plots.

Assumptions from the Langmuir model were used to develop Jovanovic Isotherm. In addition to the assumptions of the Langmuir model, the possibility of some mechanical contact between the adsorbate and adsorbent was also considered as new for the Jovanovic Isotherm. Equation (7) shows the linear form of the Jovanovic model.

(7) $$Ln\left(\frac{x}{m}\right)=Ln\;\left(q_{max}\right)-K_{J}C_{e}$$

where *C_e_* is the equilibrium concentration (in mg/L), *K*_*J*_ is constant coefficient of Jovanovic, xm is the amount of adsorbate that was adsorbed at the equilibrium stage (mg/g), and *q*_max_ is the maximum uptake of adsorbate obtained from the plot of lnxm versus *C*_*e*_.

### 2.3. UV Radiation

Since UV radiation does not have the ability to pass through glass, containers with a length of 20 cm, a width of 5 cm and a depth of 2 cm were used for the experiments. A UV lamp was installed a few millimeters away from the surface of the synthetic ASCA.

#### 2.3.1. Effect of pH

UV containers containing 100 mL of synthetic ASCA were prepared. The pH of the UV container was adjusted to 2. Next, the UV container was exposed to UV radiation with a power of 163 mW/cm^2^ at 25 °C for 15 min. After that, this experiment was repeated for pHs of 5, 9, 11 and 13. Then, the concentration of COD in all UV containers was measured.

#### 2.3.2. Effect of Temperature

Six UV containers containing 100 mL of synthetic ASCA were prepared. By adding a suitable amount of hydrochloric acid. the pH of the UV containers was adjusted to 2. Then, containers 1, 2, 3, 4, 5 and 6 were exposed to UV radiation with a power of 163 mW/cm^2^ at temperatures of 25, 35, 55, 65, 75 and 85 °C for 15 min, respectively.

#### 2.3.3. Effect of HRT

Four UV containers containing 100 mL of synthetic ASCA were prepared. By adding an appropriate amount of hydrochloric acid, the pH of the synthetic ASCA was adjusted to 2. The UV containers were then exposed to UV radiation at 163 mW/cm^2^ at 25 °C for 20 min. This experiment was repeated in HRTs at 30, 35, 50 and 80 min.

#### 2.3.4. Effect of UV Radiation

A UV container containing 100 mL of synthetic ASCA was prepared. Then, by adding an appropriate amount of hydrochloric acid, the pH of the synthetic ASCA was adjusted to 2. Next, the container was exposed to UV radiation with a power of 163 mW/cm^2^ at 25 °C for 60 min. Finally, the amount of COD of synthetic ASCA was measured. This experiment was repeated with UV radiations of 103, 118, 133, 148, and 163 mW/cm^2^.

### 2.4. Fe (VI)

#### 2.4.1. Production of Ferrate (VI)

**Figure 2:**
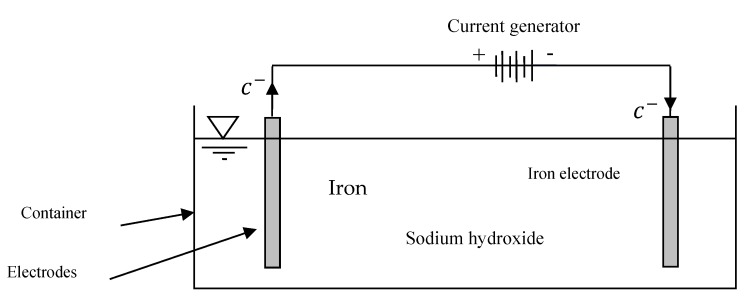
Schematic of the electrochemical cell for ferrate (VI) synthesis.

In this study, the method introduced by [32] was used to produce Fe(VI). Based on Figure 2, two rectangular iron pieces of with thicknesses of 0.63 mm and dimensions of 60 × 24 mm were used as the cathode and anode electrodes. The electrical power supplier used in this study was able to produce a DC voltage of between 1 and 24 V. The details of the Fe(VI) generator are presented in Figure 2. In this study, 14 M (56 g) sodium hydroxide solution was added to 100 mL of distilled water. Then, 100 mL of 14 M sodium hydroxide was added to the Fe(VI) generator. Subsequently, the electrodes were charged with a DC current with a voltage of 9 V and an amperage of 1 A for 30 min. As the Fe(VI) was gradually converted to Fe(III), the produced Fe(VI) solution was used in the experiments as quickly as possible.

#### 2.4.2. Effect of pH

Five 250 mL Erlenmeyer flasks containing 100 mL of synthetic ASCA were prepared. Then, a concentration of 2.53 mg/L Fe (VI) was prepared. Next, by adding a suitable amount of hydrochloric acid or sodium hydroxide, the pHs of flasks 1, 2, 3, 4 and 5 were adjusted to 2, 5, 7, 9 and 13, respectively. After that, the flasks were kept at 25 °C for 15 min.

#### 2.4.3. Effect of Fe (VI) Concentration

Four Erlenmeyer flasks with a volume of 250 mL containing 100 mL of synthetic ASCA were prepared. Then, the pH of the flasks was adjusted to 2. Next, adding a suitable amount of Fe (VI) to flasks 1, 2, 3, and 4, the concentration of Fe (VI) was adjusted to 0.09, 1.2, 2, 2.2 and 2.5 mg/L. After that, the Erlenmeyer flasks were maintained at 25 °C for 15 min.

#### 2.4.4. Effect of HRT

Four 250 mL Erlenmeyer flasks containing 100 mL of synthetic ASCA were prepared. Then, the pH of the Erlenmeyer flasks was adjusted to 2. Next, a suitable amount of Fe (VI) was added to each of the flasks to reach a Fe (VI) concentration of 2.5 mg/L. After that, flasks of 1 to 4 were kept at 25 °C for a period of 10 to 25 min.

#### 2.4.5. Effect of Temperature

Four 250 mL Erlenmeyer flasks containing 100 mL of synthetic ASCA were prepared. Then, the pH was adjusted to 2. Next, an appropriate amount of Fe(VI) was added to each of the flasks to reach a Fe(VI) concentration of 2.38 mg/L. After that, flasks of 1 to 4 were kept at a temperature of 25, 40, 50 and 60 °C, respectively, for 20 min.

### 2.5. Analytical Methods

All chemicals except ZnO nanoparticles, azithromycin and Fe(VI) were purchased from the Merck Company. ZnO nanoparticles were purchased from Sigma-Aldrich Company. Based on the information provided by this company, the size of the ZnO nanoparticles was <50 nm. Analytical grade azithromycin was purchased from Sigma, Malaysia. In this study, Fe(VI) was prepared by the electrochemical method according to the instructions described in [33]. A digital pH meter, the AZ-86502 (Taiwan) was used to measure the pH. The power of UV radiation was measured using a 340A UV meter (Taiwan). Equation (8) was used to determine the efficiency of antibiotic removal from the water in all experiments. The amount of antibiotic was determined by a COD test. The COD was measured by APHA standard method [34].

(8) $$RE=\frac{C_{1}-C_{2}}{C_{1}}\times 100$$

## 3. Results

**Figure 3:**
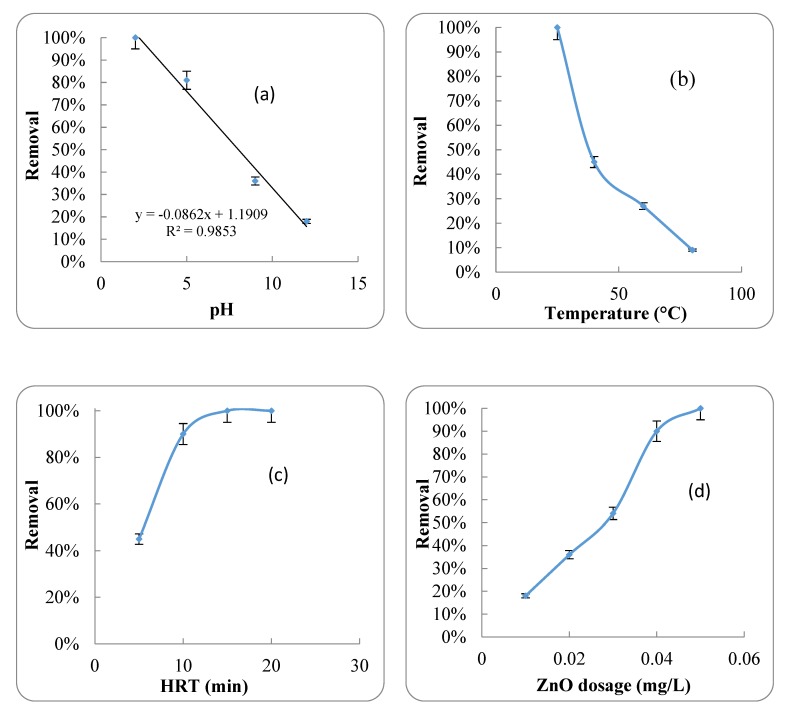
(**a**) Effect of pH changes on the removal efficiency of the antibiotic by ZnO nanoparticles; (**b**) the effects of different temperatures on antibiotic adsorption by ZnO nanoparticles; (**c**) the effect of HRT changes on the antibiotic removal efficiency by ZnO nanoparticles and (**d**) the effect of different concentrations of ZnO nanoparticles on antibiotic removal efficiency.

The effect of pH changes on the removal efficiency of the antibiotic by ZnO nanoparticles is shown in Figure 3a. As shown, pH and antibiotic removal have an inverse relationship, i.e., an increase in pH causes a decrease in antibiotic removal. The effects of different temperatures on antibiotic adsorption by ZnO nanoparticles are shown in Figure 3b. This figure shows that at lower temperatures, the antibiotic was better removed by the ZnO nanoparticles. The effect of HRT changes on the antibiotic removal efficiency of ZnO nanoparticles is shown in Figure 3c, and the effect of different concentrations of nanoparticles on antibiotic removal efficiency is shown in Figure 3d. The results showed that increasing both HRT and ZnO nanoparticle dosage had a positive effect on antibiotic removal.

**Figure 4:**
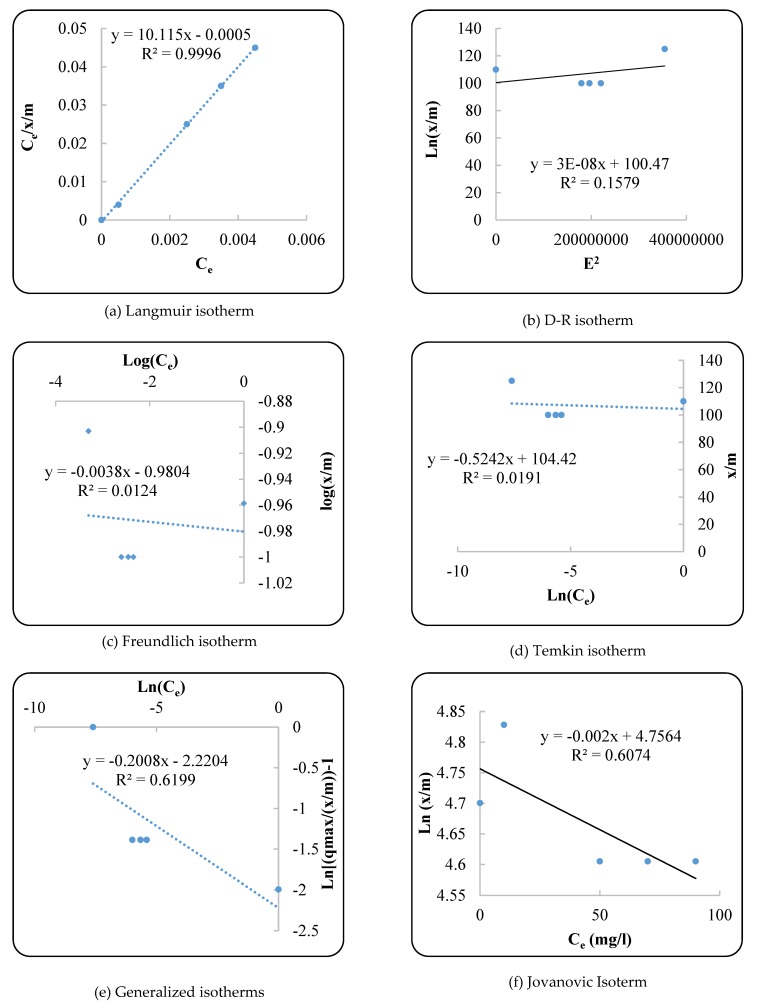
(**a**–**f**) The regression of Langmuir, D-R, Freundlich, Temkin, Generalized, and Jovian isotherms for removal of antibiotic from synthetic ASCA.

In this study, the Langmuir, Freundlich, D-R, Temkin, Genalized, and Jovian isotherms for the removal of an antibiotic from synthetic ASCA were investigated. The regression of these isotherms and their correlation coefficients are shown in Figure 4a–f.

**Figure 5:**
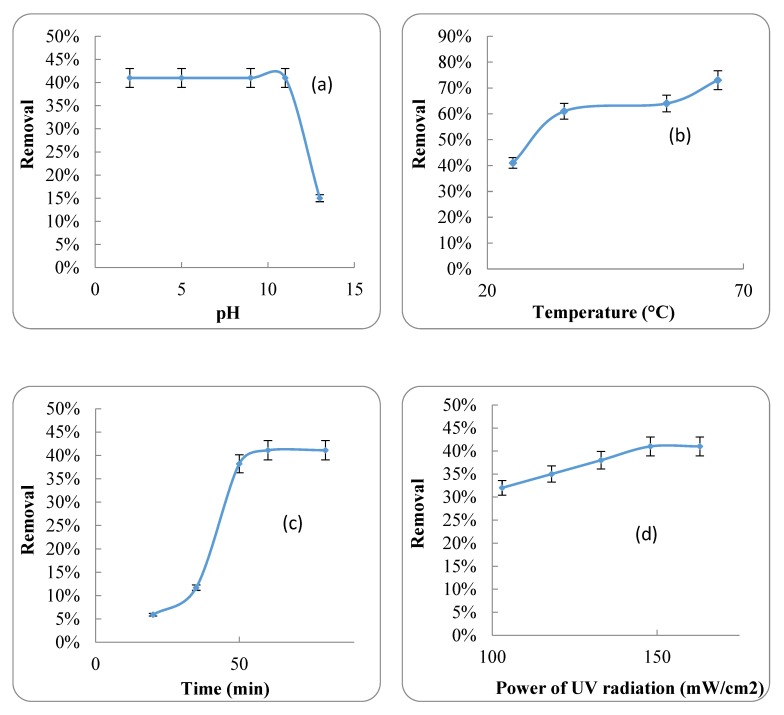
The antibiotic removal by UV radiation (**a**) The effect of different pHs; (**b**) The effect of temperature change; (**c**) The effect of HRT (**d**) The effect of UV radiation power.

The effect of different pHs, temperature, HRT and UV radiation power on antibiotic removal efficiency by UV radiation is shown in Figure 5.

**Figure 6:**
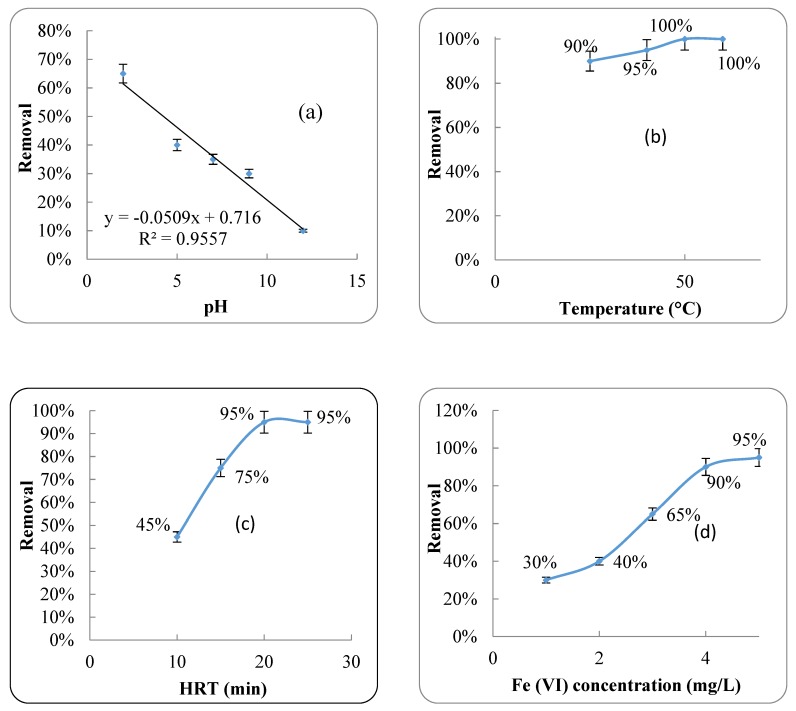
Antibiotic removal efficiency by Fe (VI); (**a**) The effect of pH; (**b**) The effect of temperature; (**c**) The effect of HRT; and (**d**) The effect of Fe(VI) concentration.

Another part of this study was related to antibiotic removal using Fe (VI); therefore, the effect of pH, temperature, HRT and Fe (VI) concentration on antibiotic removal efficiency by Fe (VI) was investigated. The results are shown in Figure 6.

## 4. Discussion

### 4.1. ZnO Nanoparticles

pH has been introduced as an effective parameter in the absorption process [35]. In acidic pH, hydrogen cations (protons) absorbs electron pairs of etheric oxygen which are between the aromatic ring and chair structure because of their empty orbitals [36]. Thus, the chair structure becomes separated from the aromatic ring and the hydroxyl group replaces it. The break-down of both bonds of etheric oxygen is dependent on the temperature and entropy of the system. Under a basic pH, the hydroxyl group of the reaction ambient can completely separate both chair-like structures from the aromatic ring. So, the aromatic ring will contain aldehyde group and both chair-like structures will have a hydroxyl group. All of the produced structures have a negative charge under a basic pH. 

As stated, the pH of the zeta point of ZnO nanoparticles is 9; the surface of ZnO may be positive in pHs below 9 and negative in pHs greater than 9 [37]. Therefore, it is possible to predicte the maximum efficiency of adsorption to be achieved under a strong acidic pH if a breakdown of etheric bonds is incomplete and the antibiotic has a negative charge.

Based on these results, by increasing the pH of ASCA, the amount of absorbed antibiotic on the ZnO nanoparticles was reduced (Figure 3a). The highest antibiotic absorption efficiency was 99.9% at a pH of 2. By increasing the pH from 2 to 5, the absorption efficiency decreased to 81%. At pH 13, only 18% of the antibiotic was absorbed. Therefore, the relationship between antibiotic adsorption efficiency and pH at 25 °C, a HRT of 15 min, an antibiotic concentration 110 mg/L and a ZnO nanoparticle concentration of 0.05 mg/1 can be described by Equation (9).

(9) $$y=\;-0.0862\;x+1.1909\;\left(R^{2}=0.98\right)$$

In this equation, y is the efficiency of the antibiotic adsorption (%) and x is the pH of the synthetic ASCA. As reported by [38], 42% to 50% of Ciprofloxacin can be removed at pHs between 7 and 10 using ZnO nanoparticles. Temperature is another effective parameter in the absorption process [39]. As shown in Figure 3b, increasing the temperature had a negative effect on the adsorption efficiency of antibiotic by ZnO nanoparticles. At 25 °C, more than 99.9% of the antibiotic was adsorbed from the ASCA by ZnO nanoparticles. However, by increasing the temperature, the adsorption efficiency decreased sharply. In addition, only 40% and 9% of antibiotic were adsorbed by ZnO nanoparticles at 40 °C and 90 °C, respectively. It can be noted that if the amount of absorption increases with increasing temperature, the process of absorption is endothermic. This is due to the increase in the mobility of the absorbed molecules and an increase in the number of active adsorption sites at higher temperatures [40]. In contrast, by increasing in temperature, the absorption efficiency decreases, which shows that the adsorption is an exothermic process. It is caused by an increase in the absorption force between a specific material and active sites on the surface of the adsorbent by temperature increase [40]. Therefore, in this study, the azithromycin adsorption process with ZnO nanoparticles was an exothermic process.

The results also prove that only one of the two etheric oxygen bonds could be broken at low temperatures, low entropies and acidic pHs. So, the electric charge of the antibiotic is negative and it can be adsorbed by the positively-charged surface of the nanoparticles under acidic conditions. It can be assumed that as the entropy of the system increases by increasing the temperature, both etheric groups may be broken, and the antibiotic would become relatively positively-charged. Therefore, the efficiency of adsorption of the antibiotic on the positively-charged surface of the ZnO nanoparticles can be increased [41,42].

The concentration of nanoparticles is also a major parameter for the efficiency of antibiotic adsorption [43]. The results showed that by increasing the concentration of ZnO nanoparticles from 0.01 to 0.05 g/L, the antibiotic adsorption efficiency increased from 18% to 99.9%. It was found that the highest adsorption efficiency was achieved when the A/P ratio was 0.00009. A smaller A/P ratio showed that the adsorbent was able to adsorb a higher amount of antibiotic. As reported by [44], the amount of absorbed antibiotic (fluoroquinolone) increased with an increase in the concentration of nanoparticles (nanoMCN@MIPs).

HRT is another important parameter for determining the efficiency of the absorption process. Several studies have been carried out on the removal of different pollutants from water or wastewater using ZnO nanoparticles [35,38,45,46], with a different HRTs, which shows that this parameter is related to the type of pollutant. Figure 3c shows the effect of HRT on the removal efficiency of antibiotics with ZnO nanoparticles. The results showed that by increasing the HRT from 5 to 10 min, the antibiotic adsorption efficiency increased greatly, i.e., from 40 to 80%. Hence, the highest antibiotic adsorption was at a HRT of 15 min, i.e., 99.9%.

#### Isotherms

To determine the rate of antibiotic adsorption on the surface of ZnO nanoparticles, isotherms such as Langmuir, Freundlich, D-R, Temkin, Genalized, and Jovian were used. The results of these isotherms are shown in Figure 4 and Table 1. Based on the results, the Langmuir equation with a correlation coefficient (R^2^) of 0.99 had the best R^2^ in comparison to other isotherms. This means that the process of absorbing the antibiotic on the ZnO nanoparticles can be correctly fitted by the Langmuir equation. The constant coefficients *q_max_* and *K_m_* in the Langmuir equation were 0.098 mg/g and −20,242.91 L/mg, respectively. In addition, the separation parameter (S.F) was calculated using Equation (10):

(10) $$S.F.\;=\frac{1}{1+K_{L}C_{0}}$$

where *C_o_* is the initial dye concentration (mg.L^−1^) and *K_L_* is related to the affinity of the binding sites. The S.F was 1, showing that the adsorption process was linear [47].

### 4.2. UV Radiation

Different amounts of antibiotic were removed at different pHs using UV radiation (Figure 5a). The results showed that the antibiotic removal efficiency was 41% when the pH was in the range of 2 to 11. By increasing the pH from 11 to 13, the efficiency of antibiotic removal was reduced from 41% to 15% using UV radiation. As reported by [48], an increase of pH causes an increase in the removal efficiency of organic compounds and hydrogen sulfide by UV radiation. Moreover, [32] found that the optimal pH for the removal of 1, 9-Dimethyl-Methylene Blue Zinc Chloride Double Salt by UV irradiation was in the range of 9.8 to 13.5.

In acidic pHs, UV light produces free radicals on the surface of the antibiotic. This means that etheric oxygen containing nonbonding electron pairs would be positive due to the loss of one electron. For this reason, the etheric oxygen gains its bonding electron and the bond between the carbon of the chair structure and the etheric oxygen would be broken. However, in basic pHs, the concentration of OH^−^ is increased. So, UV light attacks the nonbonding electron of OH^−^ instead of the nonbonding electron of the etheric oxygen. It can be concluded that OH^−^ prevents the attack of UV light upon etheric oxygen at high concentration and high pHs. Therefore, the removal efficiency of the antibiotic could be decreased. It is to be expected that the broken of etheric bond increases with an increase of temperature and entropy in the presence of UV light [42,45,46,49].

Temperature can also be considered an effective parameter on the removal of the antibiotic from the ASCA. The effect of temperature is shown in Figure 5b. Based on the results, the removal efficiencies at 25 °C and 65 °C were 41% and 73%, respectively. It can be concluded that increasing the temperature had a significant effect on antibiotic removal efficiency. In addition, increasing the HRT from 20 to 60 min had a significant effect on the removal efficiency. However, with an increase of HRT to more than 60 min, there was no significant increase in antibiotic removal (Figure 5c).

UV radiation is also an effective parameter in the removal of organic compounds such as antibiotics from water [50]. The results showed that increasing UV radiation resulted in an increase in the removal efficiency of antibiotic from the ASCA (Figure 5d). Under UV radiation of 103 mW/cm^2^, the removal efficiency was 32%. Meanwhile, by increasing the UV-radiation power to 163 mW/cm^2^, the removal efficiency reached 41%. Some other studies have shown that increasing UV power could strongly increase the pollutant removal efficacy [48]. For instance, the photo-electro-Fenton process can be used for the removal of metronidazole from wastewater. By increasing the pH, electric current intensity, UV irradiation and metronidazole concentration, the removal efficiency is increased [51].

### 4.3. Fe (VI) Oxidation Process

Advanced oxidation processes are a set of chemical treatment procedures designed to remove organic materials from water by oxidation through reactions with hydroxyl radicals. pH is one of the most important parameters affecting chemical reactions in this method [48]. The results showed that increasing the pH was an important parameter in the removal of antibiotic by the Fe (VI) oxidation process. The efficiency of antibiotic removal from wastewater by Fe (VI) at pH 2 was 65%. By increasing the pH to 12, the antibiotic removal efficiency was reduced to 10% (Figure 6a). The relationship between pH and antibiotic removal efficiency by Fe (VI) can be described using the equation y = −0.0509x + 0.716 with an R^2^ of 0.95. Several studies have reported similar results for the removal of hydrogen sulfide and organic compounds from domestic wastewater and formaldehyde from synthetic wastewater using Fe (VI) [48,52].

While Fe (VI) in acidic conditions has good potential for the removal of organic compounds from the wastewater, the cost of pH reduction is very high [53]. As calculated by [53], the cost of a pH reduction of one m^3^ of domestic wastewater using sulfuric acid is approximately 2.52$.

(11) $$\mathrm{Iron}\;\mathrm{electrode}\overset{Electrolysis+NaOH}{\to }Ferrate\left(VI\right)$$

Fe (VI) can be electrochemically produced in two forms: FeO_4_^−2^ and HFeO_4_^−^ (Equations (11) and (12)). The consumption rate of each can be calculated by Equation (12). In this equation, K_1_ and K_2_ are the constants of the consumption of HFeO_4_^−^ and FeO_4_^−2^.

(12) $$K\left[Ferrate\right]=K_{1}\left[HFeO_{4}^{-}\right]+K_{2}\left[FeO_{4}^{2-}\right]\;$$

The constant rates of consumption of FeO_4_^−2^ and HFeO_4_^−^ are 1.24 × 10^2^ M/s and 8.41 × 10^2^ M/s, respectively. As a result, the reaction rate between the antibiotic and HFeO_4_^−^ was higher than that of FeO_4_^−2^. Therefore, HFeO_4_^−^ had a significant effect on antibiotic removal from the wastewater. Since in acidic conditions, the dominant formation of Fe (VI) is HFeO_4_^−^, antibiotic removal under acidic conditions was better than under alkaline condition. 

The results showed that changing the temperature from 25 °C to 60 °C had little effect on the removal efficiency by the Fe (VI) oxidation process (Figure 6b). Increasing the temperature from 25 °C to 60 °C could lead to an increase in the antibiotic removal efficiency from 90% to 100%. Increasing HRT from 10 to 20 min increased the removal efficiency from 45% to 95% (Figure 6c). However, there was no significant effect on antibiotic removal by Fe (VI) when the HRT was increased to more than 20 min.

The concentration of Fe(VI) is also another important parameters in the removal of antibiotics from synthetic wastewater [50]. Increasing the concentration of Fe (VI) from 1 to 4 mg/L increased the antibiotic removal efficiency. However, by increasing the Fe (VI) concentration to more than 4 mg/L, the antibiotic removal efficiency was not significantly increased (Figure 6d). Reporting the optimal A/P instant of the Fe (VI) concentration is a better strategy for industrial applications. The results showed that the optimal A/P was 0.011 to 0.012, and that Fe(VI) is a suitable method to remove azithromycin from wastewater. It is important that this method be used to remove other common antibiotics in real samples of wastewater to properly understand all of its advantages and disadvantages. Then, the Fe(VI) oxidation process can be applied on a large scale to remove a mixture of different antibiotics.

## 5. Conclusions

In this study, the removal of the antibiotic azithromycin from synthetic wastewater by three different methods, namely Fe (VI) oxidation, UV irradiation and absorption by ZnO nanoparticles, was investigated. It was found that the efficiencies of antibiotic removal by Fe (VI) oxidation, UV irradiation and absorption by ZnO nanoparticles were 100%, 73%, and 100%, respectively. In addition, reducing the pH led to a sharp increase in the removal efficiency by the Fe (VI) oxidation process. By increasing HRT up to 20 min, the antibiotic removal efficiency increased, while higher HRTs had no significant effect on removal efficiency. Also, the effect of temperature on the removal efficiency was low using the Fe (VI) oxidation process. The optimal ratio of Fe (VI) concentration to the initial concentration of antibiotic was 0.011 and 0.012. Increasing the temperature to 60 °C had a positive effect on the removal efficiency by UV radiation and ZnO nanoparticles. For future studies, the investigation of a combination of the Fe (VI) oxidation process, UV radiation and ZnO nanoparticles is recommended.

## Figures and Tables

**Table 1:** Constant coefficient of isotherms.

Jovian			Genalized			D-R			Temkin			Freundlich			Langmuir		
q_max_	K_j_	R^2^	N	K_G_	R^2^	q_max_	β	R^2^	K_T_	B_1_	R^2^	k_f_	n	R^2^	q_m_	K_e_	R^2^
116.33	0.002	0.6074	0.2008	0.10856	0.6199	4.3 × 10^43^	3 × 10^−8^	0.15	3.11 × 10^−87^	0.5242	0.0191	0.99	−263.2	0.012	0.098	−20242.91	0.99

R^2^ = statistical measure of how the data are fitted; q_m_ and K_e_ = Langmuir constants; n and K_f_ = constant coefficients of the Freundlich equation; In Temkin Isotherm, K_T_ = equilibrium binding constant (in L/mg) corresponding to maximum binding energy and the value increased with an increase in temperature for both the adsorbents, which is suggestive of the corresponding increase of maximum binding energy. Constant B_1_ = is related to the heat of adsorption; In D-R isotherm, q_max_ = maximum amount of adsorbate that can be adsorbed on the adsorbent; β = constant related to energy. In Generalized isotherm, K_G_ = saturation constant (in mg/L); N = cooperative binding constant; q_max_ = maximum adsorption capacity of the adsorbent (in mg/g); In Jovian Isotherm, *K*_*J*_ = constant coefficient of Jovanovic, and *q*_max_ = maximum uptake of adsorbate.
